# Satellite DNA Modulates Gene Expression in the Beetle *Tribolium castaneum* after Heat Stress

**DOI:** 10.1371/journal.pgen.1005466

**Published:** 2015-08-14

**Authors:** Isidoro Feliciello, Ivana Akrap, Đurđica Ugarković

**Affiliations:** 1 Department of Molecular Biology, Ruđer Bošković Institute, Zagreb, Croatia; 2 Dipartimento di Medicina Clinica e Chirurgia, Universita’ degli Studi di Napoli Federico II, Napoli, Italy; University of Cambridge, UNITED KINGDOM

## Abstract

Non-coding repetitive DNAs have been proposed to perform a gene regulatory role, however for tandemly repeated satellite DNA no such role was defined until now. Here we provide the first evidence for a role of satellite DNA in the modulation of gene expression under specific environmental conditions. The major satellite DNA TCAST1 in the beetle *Tribolium castaneum* is preferentially located within pericentromeric heterochromatin but is also dispersed as single repeats or short arrays in the vicinity of protein-coding genes within euchromatin. Our results show enhanced suppression of activity of TCAST1-associated genes and slower recovery of their activity after long-term heat stress relative to the same genes without associated TCAST1 satellite DNA elements. The level of gene suppression is not influenced by the distance of TCAST1 elements from the associated genes up to 40 kb from the genes’ transcription start sites, but it does depend on the copy number of TCAST1 repeats within an element, being stronger for the higher number of copies. The enhanced gene suppression correlates with the enrichment of the repressive histone marks H3K9me2/3 at dispersed TCAST1 elements and their flanking regions as well as with increased expression of TCAST1 satellite DNA. The results reveal transient, RNAi based heterochromatin formation at dispersed TCAST1 repeats and their proximal regions as a mechanism responsible for enhanced silencing of TCAST1-associated genes. Differences in the pattern of distribution of TCAST1 elements contribute to gene expression diversity among *T*. *castaneum* strains after long-term heat stress and might have an impact on adaptation to different environmental conditions.

## Author Summary

Non-coding repetitive DNAs constitute a considerable portion of most eukaryotic genomes and their function is intensively investigated. Here we analyse a gene-regulatory role for tandemly repeated satellite DNA which is the major building element of pericentromeric and centromeric heterochromatin in many eukaryotes. We use as a model system the beetle *Tribolium castaneum* which has a major satellite DNA preferentially located in pericentromeric heterochromatin but satellite repeats are also dispersed in the vicinity of protein-coding genes within euchromatin. Our results demonstrate for the first time the role of satellite DNA in the modulation of protein-gene expression and reveal the molecular mechanism of their gene-regulatory activity. The influence of satellite DNA on neighbouring genes is epigenetic in nature and is induced by specific changes in the environment such as long-term heat stress. Based on this, the impact of satellite DNAs on adaptation to different environmental conditions as well as their role in the evolution of gene regulatory networks is proposed.

## Introduction

Non-coding repetitive DNA includes diverse types of sequences which comprise a considerable portion of most eukaryotic genomes and their function is intensively investigated. In particular, mobile transposable elements are predicted to be a source of noncoding material and to influence evolution of gene regulatory networks [1]. Effects on gene regulation of another abundant class of non-coding repetitive DNA, tandemly repeated satellite DNA, was proposed based on the presence of promoter elements and transcription factor binding sites within some satellite sequences, as well as due to their transcriptional activity [2–4]. However, experimental studies revealing the influence of satellite DNA on the activity of genes are still lacking. This is mostly due to the specific organization of satellite DNAs which in the form of long arrays constitute gene-poor heterochromatin located in pericentromeric, centromeric and/or telomeric regions of chromosomes [5,6]. There are however several examples of satellite DNAs present not exclusively within pericentromeric heterochromatin but also dispersed as single repeats or short arrays in the vicinity of genes within euchromatin, such as the 1.688 *Drosophila melanogaster* satellite DNA [7] and two satellite DNAs from the beetle *Tribolium castaneum* [8,9]. Such dispersed organizational patterns might be formed due to intrastrand homologous recombination within satellite arrays [10], resulting in the formation of extrachromosomal circles [11] and their subsequent insertion throughout the genome [8,9]. Although satellite DNAs are mainly embedded in tightly packed heterochromatin which is perceived as transcriptionally silent, low expression of these sequences was reported in vertebrates, invertebrates and plants [2,12]. Satellite DNA transcripts were shown to be processed into small interfering RNAs (siRNAs), 20–25 nt in length, which are involved in the epigenetic process of heterochromatin formation in diverse organisms such as fission yeast *Schizosaccharomyces pombe*, nematodes, flys and plants [13–16]. The transcription of satellite DNAs is usually gender or stage specific and is often associated with differentiation and development or with certain stress conditions, particularly heat stress. In *Arabidopsis*, after exposure to prolonged heat stress, transcription of satellite DNA is significantly increased and is accompanied by heterochromatin decondensation [17,18]. Expression of human satellite III which is not detected under standard conditions is also induced by heat stress [19,20]. Increasing evidence on the reorganization of heterochromatin elicited by heat stress and other stress treatments could indicate a possible role for satellite DNA in a general stress response activated in cells to cope with harmful conditions [4].

The insect *Tribolium castaneum* has a major satellite DNA TCAST1 which comprises 35% of the genome and encompasses the pericentromeric regions of all chromosomes [21], but is also dispersed within euchromatin [8]. The expression of the satellite DNA TCAST1 is strongly induced by heat shock and proceeds in the form of long double-strand transcripts which are rapidly processed into 21–30 nt siRNAs [22]. Enhanced TCAST1 satellite DNA expression is accompanied by an increase in repressive epigenetic modifications of histones at satellite DNA regions in heterochromatin [22]. Now we propose that single repeats or short stretches of TCAST1 satellite DNA which are dispersed in the close vicinity of protein-coding genes within euchromatin are also subjected to such epigenetic changes induced by TCAST1 siRNAs. Since heterochromatin influences genes located in the vicinity [23,24], we expect that heterochromatic marks which might be established on dispersed TCAST1 repeats could affect nearby genes, most probably by inducing their silencing. The aim of the present study was to test the proposed hypothesis of a potential gene-regulatory role of TCAST1 satellite DNA. Our approach was to analyse insertion and copy number polymorphism of dispersed TCAST1 satellite repeats among *T*. *castaneum* strains and to examine if the presence/absence of TCAST1 repeats is correlated with the activity of adjacent genes under standard and heat stress conditions. In addition, we examined the epigenetic state of regions flanking dispersed TCAST1 satellite elements and correlated it with the expression level of nearby genes. Our results reveal for the first time a role for satellite DNA in the modulation of gene expression under the specific condition of long-term heat stress. In addition, it was shown that transient RNAi based establishment of heterochromatin at dispersed satellite elements and its spreading into proximal regions represents a mechanism responsible for the satellite DNA-mediated silencing of nearby genes.

## Results

### Detection of dispersed TCAST1 elements polymorphic among strains

Bioinformatic analysis of *T*. *castaneum* genomic data revealed 68 elements homologous to the major TCAST1 satellite DNA, which are dispersed in the vicinity of protein coding genes [8]. There are two types of TCAST1-like elements: TCAST1 satellite-like elements in the form of partial or tandemly arranged monomers and TCAST1 transposon-like elements with a complex unit that resembles a non autonomous DNA transposon. In order to test the potential gene-regulatory role of dispersed TCAST1 elements we first analysed their insertion polymorphism among ten *T*. *castaneum* strains which originate from diverse geographic locations characterized by distinct environments. Polymorphism analysis was performed for 32 out of a total of 68 dispersed TCAST1 elements, 19 transposon-like and 13 satellite-like elements. For the remaining elements, flanking sequences were either not well characterized in GenBank or contained other repetitive sequences which made construction of single locus-specific primers impossible. The polymorphisms were associated with the following TCAST1 satellite-like elements: element 2 located within the first intron of a probable *Ser/Thr kinase* gene (GenBank accession number: 661947), element 12 located within the fifth intron of a *putative uncharacterized protein* gene (GenBank accession number: 100141521), element 21 in the second intron of *WD repeat containing protein 47* gene (GenBank accession number: 657535) and element 46 located at an intergenic region: 5820 bp from 3’ end of a *putative uncharacterized protein* gene (GenBank accession number 657017) and 7000 bp from 5’ end of *ribonucleoside-diphosphate reductase* gene (GenBank accession number 657098). Schematic representation of genes associated with polymorphic TCAST1 elements 2, 12, 21 and 46 is shown on S1 Fig.

**Fig 1:**
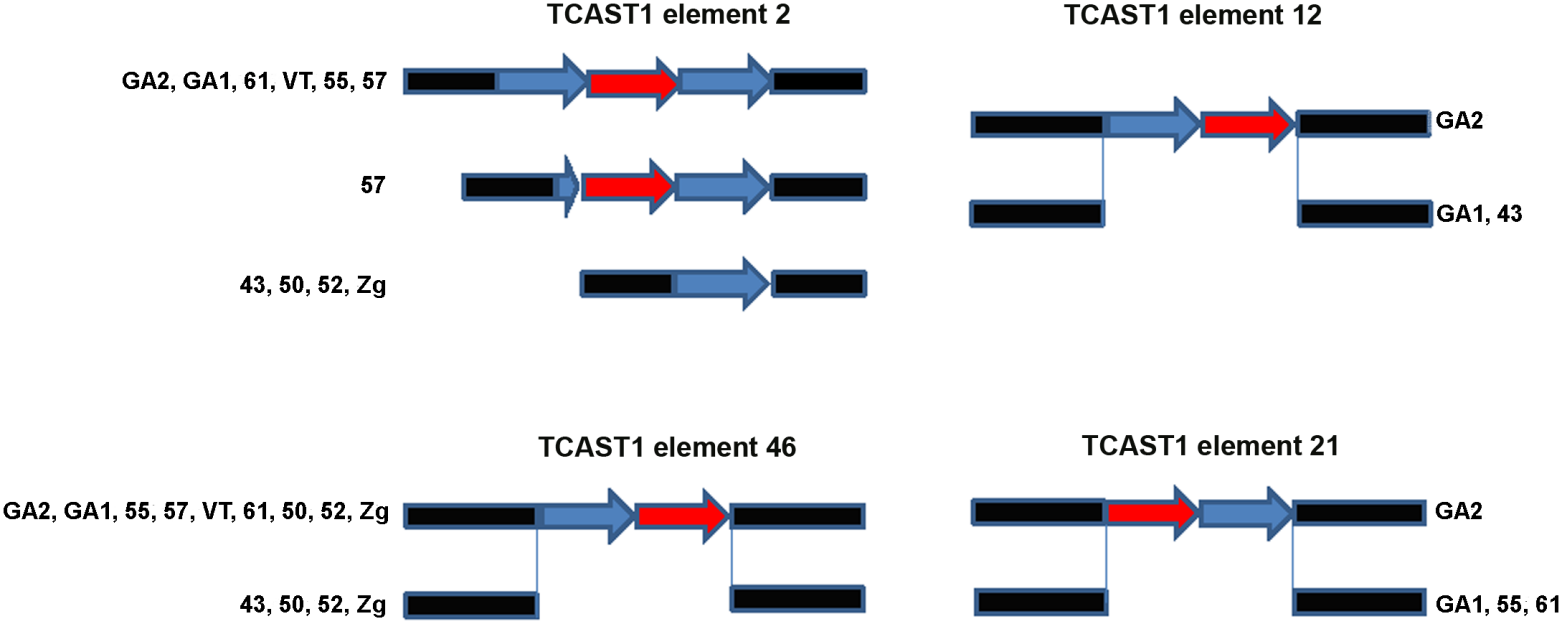
Schematic representation of dispersed TCAST1 elements 2, 12, 46 and 21 in *T*. *castaneum* strains. Blue and red arrows represent repeats belonging to subfamilies Tcast1a and Tcast2b, respectively, while flanking sequences are shown in black.

Polymorphism of the TCAST1 element 2 is due to a difference in copy number of satellite repeats among *T*. *castaneum* strains: GA1, 61, 55, VT and GA2 containing a homozygous trimer, 43, 50, 52 and Zg contain a homozygous monomer, while strain 57 has mostly homozygous dimers but in rare individuals a trimer is also detected (Fig 1). Sequence analysis reveals that trimers and dimers are composed of two related repeats which belong to TCAST1 subfamilies Tcast1*a* and Tcast1*b* [20], respectively. TCAST1 element 12 is present as a homozygous dimer of two partial Tcast1a and Tcast1b monomers in the genome of GA2 strain and is completely absent from strains GA1 and 43 (Fig 1). TCAST1 element 21 in the form of a homozygous dimer is present in GA2 strain while strains GA1, 61 and 55 reveal its complete absence (Fig 1). TCAST1 element 46 in the form of a homozygous dimer is found in strains GA2, 55, 57, VT, 61, GA1, while it is completely absent in strain 43. Strains 50, 52 and Zg, due to heterozygosity, have both forms present (Fig 1).

### Influence of TCAST1 elements on gene expression under standard conditions

To test the potential role of dispersed TCAST1 elements in the regulation of nearby genes we analysed expression of genes associated with TCAST1 elements that showed insertion polymorphism among *T*. *castaneum* strains. The purpose of the analysis was to determine if there is any difference in the expression of genes with respect to the presence or absence of TCAST1 elements in their neighbourhood (genes associated with elements 12, 21 and 46) or with respect to different copy number of repeats within particular TCAST1 element (gene associated with element 2). The gene associated with element 12 was expressed at an extremely low level in all strains and was eliminated from further analysis.

**Fig 2:**
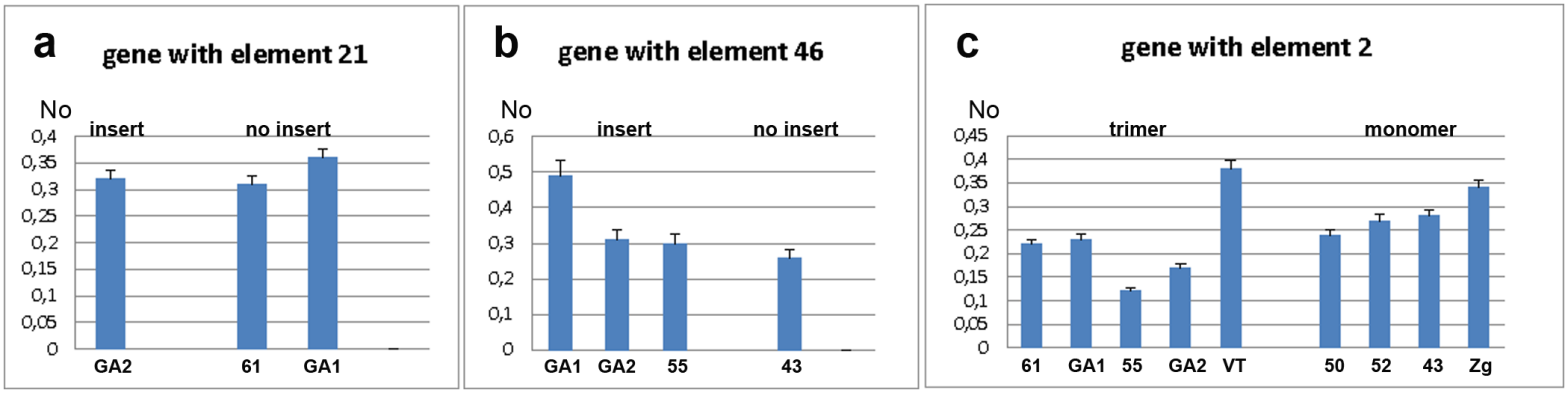
Analysis of gene expression measured by qRT-PCR under standard physiological conditions. **a)** Expression of gene associated with element 21 in strains with (GA2) and without (61, GA1) element 21. **b**) Expression of gene associated with element 46 in strains with (GA2, GA1 and 55) and without (43) element 46. **c**) Expression of gene associated with TCAST1 element 2 in strains where element 2 is in the form of trimer and monomer. No represents normalized average No value for each gene. Columns show average of two different qRT-PCR experiments performed in triplicate and error bars represent standard deviations.

The expression of the *WD repeat containing protein 47* gene was analysed by qPCR using primers designed for exons 4 and 5 (S1 Fig). Expression was analysed in strains with (GA2) and without (GA1 and 61) the gene-associated TCAST1 element 21 (Fig 2a). The mean No value (see Materials and Methods, section Quantitative real-time PCR analysis) of the gene in GA2 strain was 0.3205 ± 0.0145 relative to the mean No value of 0.3350 ± 0.0303 in the strains GA1 and 61 (P = 0.2671), as determined by the unpaired t-test, indicating no significant difference in expression between the two groups of strains. However, analysis of strain variation using the one-way ANOVA statistical test showed that genetic background plays a significant role in gene expression (P = 0.0448). Moreover, it seems that genetic background is dominating over the effect of the repetitive insert and at our level of statistical power this enables a firm conclusion regarding the influence of TCAST1 inserts on gene expression. Expression analysis of the *ribonucleoside-diphosphate reductase* gene in strains with (GA2, GA1 and 55) and without (43) the gene-associated TCAST1 element 46 revealed no significant difference in expression between the two groups of strains (Fig 2b; P = 0.1604). As in previous case, strain variation was shown to contribute significantly to gene expression (P = 0.0049), as determined by the one-way ANOVA statistical test.

Analysis of expression of a probable *Ser/Thr kinase* gene was performed in strains which contained the gene-associated TCAST1 element 2 as a trimer (GA1, 61, 55, GA2 and VT) and as a monomer (43, 52, 50, Zg), using primers designed for exons 1 and 2 as shown in S1 Fig. There was substantial variation in gene expression within strains containing a trimer and to a lesser extent within strains containing a monomer (Fig 2c). The mean No values for the gene associated with trimer and monomer were 0. 2345 ± 0.03197 and 0.2655 ± 0.01703, respectively, with a P value of 0.403, as determined by the unpaired t-test. The result shows no significant difference in the average expression of gene associated with trimer and monomer however using the one-way ANOVA statistical test again reveals that strain variation has a significant impact on gene expression (P = 0.0004).

These results reveal that under standard physiological conditions, when only influence of insert is considered, TCAST1 elements do not exhibit a significant effect on the expression of neighbouring genes. However, as strain variation is a significant and dominant factor in gene expression, we cannot make any firm conclusion about the influence of TCAST1 on gene expression.

### Influence of TCAST1 elements on gene expression after heat stress

#### a) Expression of genes associated with polymorphic TCAST1 elements

Heat stress (HS) influences expression of TCAST1 satellite DNA in *T*. *castaneum* and affects heterochromatin structure [22]. To check if dispersed TCAST1 elements affect the expression of neighbouring genes under heat stress conditions, we analysed expression of genes associated with elements 2, 21 and 46 in different *T*. *castaneum* strains after temperature stress at 40°C. A short heat stress of 2 h, as well as prolonged heat stress of up to 8 hrs, resulted in no significant change in the expression of tested genes (S2 Fig). To test if long-term heat stress has any influence on gene expression, we subjected adults of *T*. *castaneum* to 40°C for 24 hours, followed by recovery at 25°C. Under such conditions survival of adult beetles of all tested populations was almost 100%. The expression of TCAST1 satellite DNA was also checked after long-term heat stress and the results revealed increased expression reaching a maximum at 1 hour in the recovery period with the level of TCAST1 transcripts three times higher relative to the control (S3 Fig).

**Fig 3:**
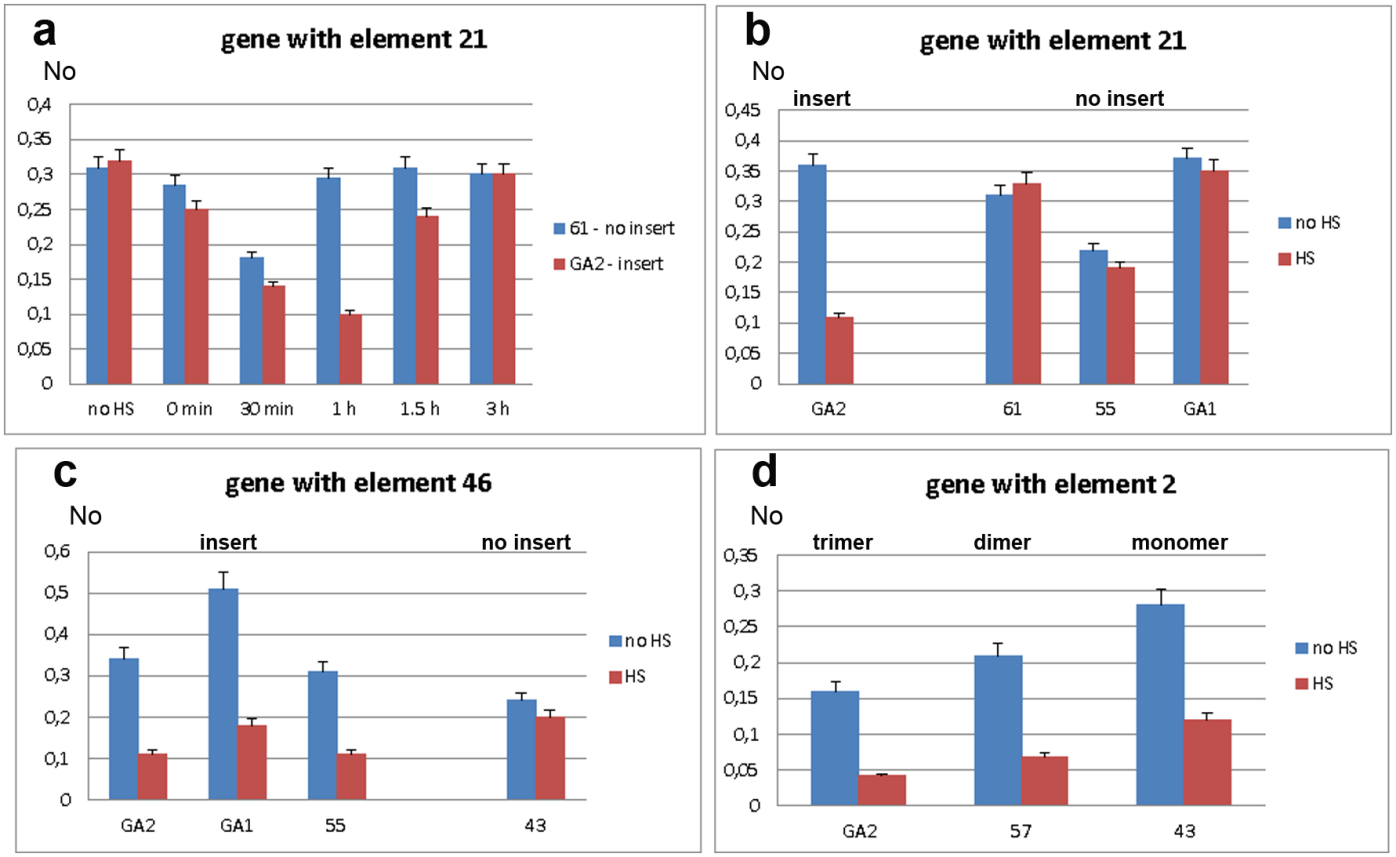
Expression of genes associated with polymorphic TCAST1 elements after heat stress. **a)** Dynamics of expression of gene associated with element 21 in strain GA2 (red) and of the same gene without element 21 in strain 61 (blue) under standard conditions (no HS), immediately after long-term heat stress and at 30 min, 1 hr, 1.5 hrs and 3 hrs of recovery period. Expression of genes associated with: **b)** element 21 in strains with (GA2) and without (61, 55, GA1) insert, **c)** element 46 in strains with (GA2, GA1 and 55) and without (43) insert, **d)** element 2 in the form of trimer (GA2 strain), dimer (57 strain) and monomer (43 strain). Blue columns in **b**, **c** and **d** represent expression at standard conditions, while red columns represent expression at 1 hour of recovery after long-term heat stress. No represents normalized average No value for each gene. Columns show average of two different qRT-PCR experiments performed in triplicate and error bars represent standard deviations.

We first assessed expression of *WD repeat containing protein 47* gene in strains GA2 and 61, with and without the associated TCAST1 element 21, respectively. Gene expression was measured immediately after heat stress and after 30 min, 1 hr, 1.5 hrs and 3 hrs of recovery (Fig 3a). In both strains, gene expression was slightly reduced immediately after heat stress and continued to decrease reaching approximately 60% and 50% of control activity in 61 and GA2 strain, respectively, after 30 min of recovery. However, after 1 hr of recovery, gene expression in strain GA2 continued to decrease being 3 times lower relative to the control, while expression in strain 61 returned to a level almost identical to the control. Approximately 70–80% recovery of gene expression in strain GA2 was observed after 1.5 hrs and almost complete recovery is observed after 3 hrs (Fig 3a). The difference in the expression of *WD repeat containing protein 47* gene in strains 61 and GA2 at 1 hr of recovery after long-term heat stress was confirmed using qPCR with a gene TaqMan probe (Fig 3b). The gene expression at 1 hr of recovery after HS was also checked in strains 55 and GA1 which, like strain 61, lack element 21 (Fig 3b). Strains 61, GA1 and 55 exhibit no significant difference in gene expression at 1 hr of recovery after HS relative to the control without HS (P values >0.4), as determined by the unpaired t-test. In contrast, in the GA2 strain the level of expression at 1 hr of recovery after HS is 3.1 times lower relative to the control (P<0.0001). The results indicate an influence of TCAST1 element 21 on the expression of an associated gene during heat stress response: the satellite element suppresses activity and slows down the kinetics of gene expression recovery after heat stress. In strains without TCAST1 element 21 such as 55, GA1 and 61, the suppression of gene activity is lower and complete recovery of gene expression occurs 1 hr after HS, while expression of the same gene with inserted TCAST1 satellite element in GA2 strain is completely recovered 3 hrs after HS.

We analysed the activity of the *ribonucleoside-diphosphate reductase* gene in strains GA2, GA1 and 55 which have the TCAST1 element 46 located at the 5’ end of the gene as well as in strain 43 where element 46 is completely absent. After long-term heat stress and a recovery period of 1 hr, expression of the tested gene revealed no significant downregulation in strain 43 (P = 0.0856), while in strains GA2, GA1 and 55 the downregulation is 3.4 x (P = 0.0001), 2.8 x (P = 0.0041) and 3.0 x (P = 0.0089), respectively, relative to the control without HS (Fig 3c), and reaches control level after 3 hrs. Therefore, the results were very similar to those for the gene associated with element 21: in both cases TCAST1 elements influence gene expression after long-term heat stress by repressing gene activity and slowing down the kinetics of gene expression recovery.

Next we analysed the activity of the gene associated with TCAST1 element 2 in strains GA2, 57 and 43. In strain GA2 element 2 is in the form of a trimer while in strains 57 and 43 it is in the form of a dimer and monomer, respectively. After a long-term heat stress of 24 hrs and recovery period of 1 hr, the gene associated with element 2 was downregulated in all strains (Fig 3d). The downregulation was 3.8 x (P = 0.0055) relative to the level before HS for the gene associated with satellite trimer, 3.1 x (P = 0.0075) and 2.4 x (P = 0.0047) for the same gene associated with dimer and monomer, respectively. The P values in all cases were calculated using the unpaired t-test.

#### b) Expression of genes associated with nonpolymorphic TCAST1 elements

**Fig 4:**
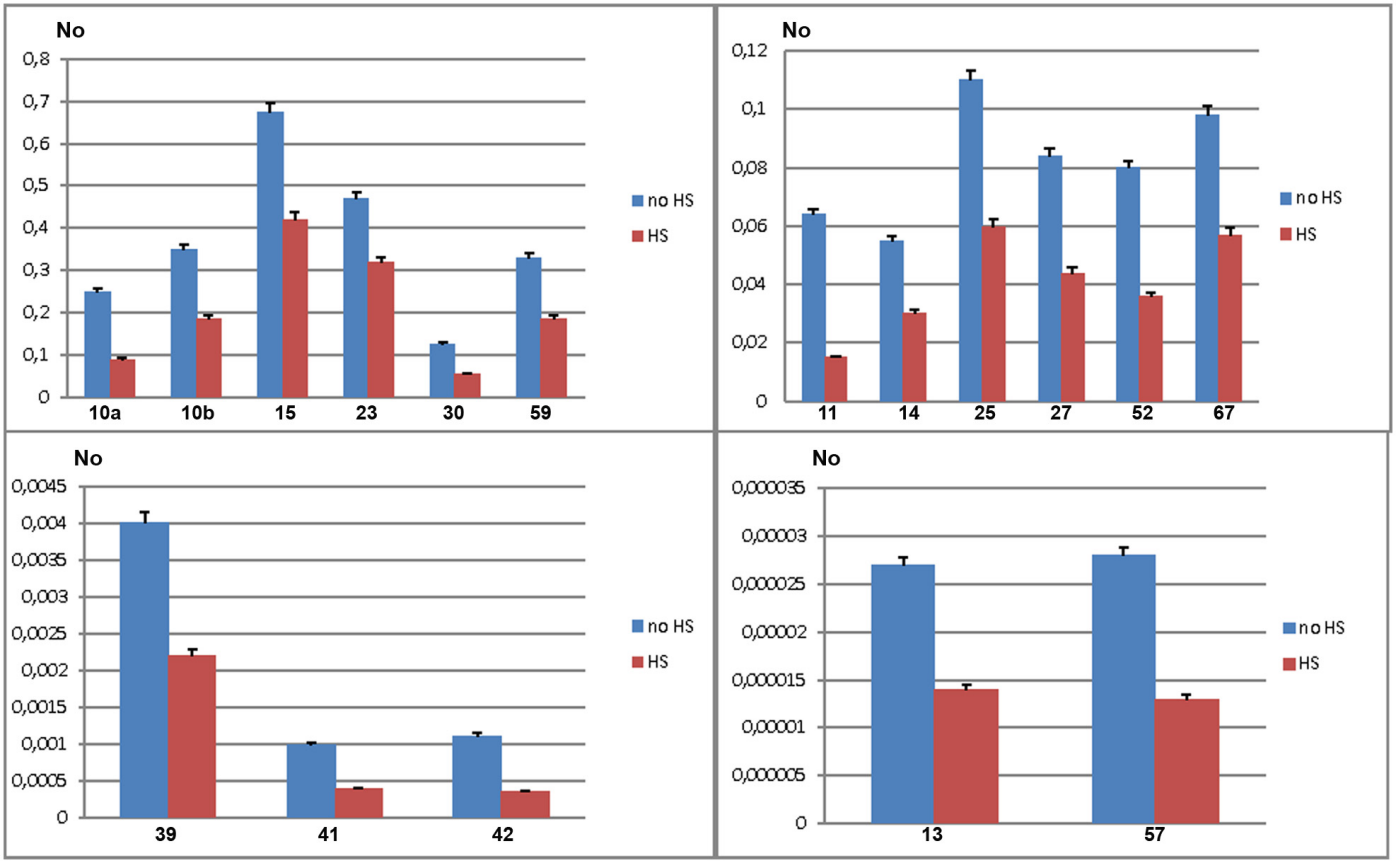
Expression of genes associated with nonpolymorphic TCAST1 elements after heat stress. Elements 10, 11, 13, 14, 15, 23, 25, 27, 30, 39, 41, 42, 52, 57, 59 and 67 in population GA2 at standard conditions (blue) and at 1 hour of recovery after long-term heat stress (red). No represents normalized average No value for each gene. Columns show average of two different qRT-PCR experiments performed in triplicate and error bars represent standard deviations.

To validate gene regulatory effect of TCAST1 satellite elements revealed by polymorphic elements 2, 21 and 46, we expanded our analysis to genes associated with TCAST1 elements which exhibited no insertion polymorphism (S1 Table). After exclusion of pseudogenes and genes with a very low level of expression, 17 genes were suitable for expression analysis. Of these, six genes were associated with TCAST1 satellite-like elements, carrying them in introns (elements 41, 59 and 67) or at 3’ sites (elements 14, 15 and 30) while eleven genes were associated with complex TCAST1 transposon—like elements which were either located within introns (elements 11, 13, 23, 27, 52, 57) or at intergenic regions (10, 25, 39, 42). Expression of genes was examined at 1 hr of recovery after long-term HS at 40°C in the GA2 strain and the results revealed significant downregulation relative to the control without HS for all 17 genes (Fig 4). The level of downregulation with statistical significance expressed in P values, calculated by the unpaired t-test, is listed in Table 1 for all tested genes associated with TCAST1 elements, either polymorphic or nonpolymorphic. TCAST1 elements are classified as satellite-like and transposon-like elements and for each of them the position relative to the associated gene and distance from the transcription start site (TSS) of the associated gene is indicated as well as the number of TCAST1 satellite monomers contained within each dispersed element (Table 1). *T*. *castaneum* strains used in expression analyses are also indicated in Table 1.

**Table 1:** List of genes (NCBI-GeneIDs) associated with TCAST1 elements and their level of suppression after long-term heat stress in different strains of *T*. *castaneum*. Types of dispersed elements with a number of TCAST1 monomers within each element, positions of TCAST1 elements relative to the genes and their distance from TSS are indicated. The P values were calculated using the unpaired t-test.

ID of gene associated with TCAST1 element	TCAST1 element	Beetle strain	Position of TCAST1 relative to gene	Distance of TCAST1 from TSS (kb)	TCAST1 element type—(number of TCAST1 monomers)	The level of gene suppression after HS (x) and significance (P- values)
661947	2	43	intron	18	Satellite (0.9)	2.4 (0.0047)
656290	14	GA2	3'	9	Satellite (0.7)	1.8 (0.0009)
658603	15	GA2	3'	20	Satellite (0.6)	1.7 (0.0050)
656976	30	GA2	3'	39	Satellite (0.6)	1.6 (0.0046)
663231	59	GA2	intron	1.5	Satellite (1.4)	1.9 (0.0111)
661207	67	GA2	intron	20	Satellite (1.0)	1.8 (0.0182)
661947	2	57	intron	18	Satellite (2.0)	3.1 (0.0075)
657535	21	GA2	intron	12	Satellite (1.7)	3.1 (0.0001)
654938	41	GA2	intron	5	Satellite (2.0)	2.6 (0.0014)
657098	46	GA2	5'	7	Satellite (2.0)	3.4 (0.0001)
657098	46	GA1	5'	7	Satellite (2.0)	2.8 (0.0041)
657098	46	55	5'	7	Satellite (2.0)	3.0 (0.0089)
661947	2	GA2	intron	18	Satellite (2.8)	3.8 (0.0055)
655561	10	GA2	3'	35	Transposon-like (1.2)	2.4 (0.0009)
655640	10	GA2	5'	14	Transposon-like (1.2)	2.0 (0.0219)
655011	11	GA2	intron	28	Transposon-like (1.2)	4.0 (0.0071)
660195	13	GA2	intron	8	Transposon-like (1.0)	2.0 (0.0219)
656174	23	GA2	intron	80	Transposon-like (1.2)	1.5 (0.0420)
659233	25	GA2	3'	13	Transposon-like (1.3)	1.9 (0.0098)
658463	27	GA2	intron	8	Transposon-like (0.8)	2.0 (0.0109)
100142507	39	GA2	5'	7	Transposon-like (1.2)	1.9 (0.0095)
662058	42	GA2	3'	14	Transposon-like (1.2)	3.1 (0.0064)
657778	52	GA2	intron	32	Transposon-like (1.1)	2.4 (0.0035)
655916	57	GA2	intron	8	Transposon-like (1.1)	2.1 (0.0196)

The data reveal an average 1.90 ± 0.28 x downregulation of genes associated with TCAST1 satellite-like elements composed of a full or partial monomer. The downregulation occurs irrespective of the position of TCAST1 satellite element relative to the gene, either at intergenic regions or within introns. The level of downregulation shows statistically insignificant, negative correlation (Pearson correlation coefficient = -0.5076, P = 0.3826) for the distance of elements from the TSS within the tested range from 1.5 kb up to 39 kb. TCAST1 satellite-like elements which contain a dimer exhibit a stronger effect on associated genes relative to the previous group, with an average 3.00 ±0.32 x downregulation, while the effect of a trimer is even stronger (Table 1), indicating a positive correlation between TCAST1 copy number and the level of gene downregulation (correlation coefficient = 0.7487, P = 0.0127).

TCAST1 transposon-like elements contain approximately a single TCAST1 monomer and genes associated with them show an average downregulation of 2.30 ± 0.69 which is not significantly different from the downregulation exhibited by a satellite monomer (P = 0.1681) or dimer (P = 0.0586), as determined by the unpaired t-test. The influence is similar on genes located 5’ or 3’ relative to the TCAST1 transposon-like element, as shown for the two genes flanking element 10 (Table 1). TCAST transposon-like elements are located within 7–35 kb from the TSS with the exception of a single element at 80 kb distance. The level of gene expression downregulation does not seem to be significantly affected by the distance of the elements from the TSS (correlation coefficient = -0.1984, P = 0.557). However, the lowest level of 1.5 x gene expression downregulation is observed for the most distant element (80 kb from TSS) indicating that at such distances effect of TCAST elements on gene downregulation might be reduced.

In conclusion, both TCAST1 satellite-like and TCAST1 transposon-like elements affect expression of associated genes during the recovery period of HS. The level of gene downregulation is positively correlated with the number of TCAST1 repeats within the elements, but it is not influenced by the position of TCAST1 element relative to the gene (5’ or 3’ end, intron) or by the distance from the associated gene, at least up to 35–40 kb from the TSS.

### Mechanism of TCAST1- mediated gene regulation

**Fig 5:**
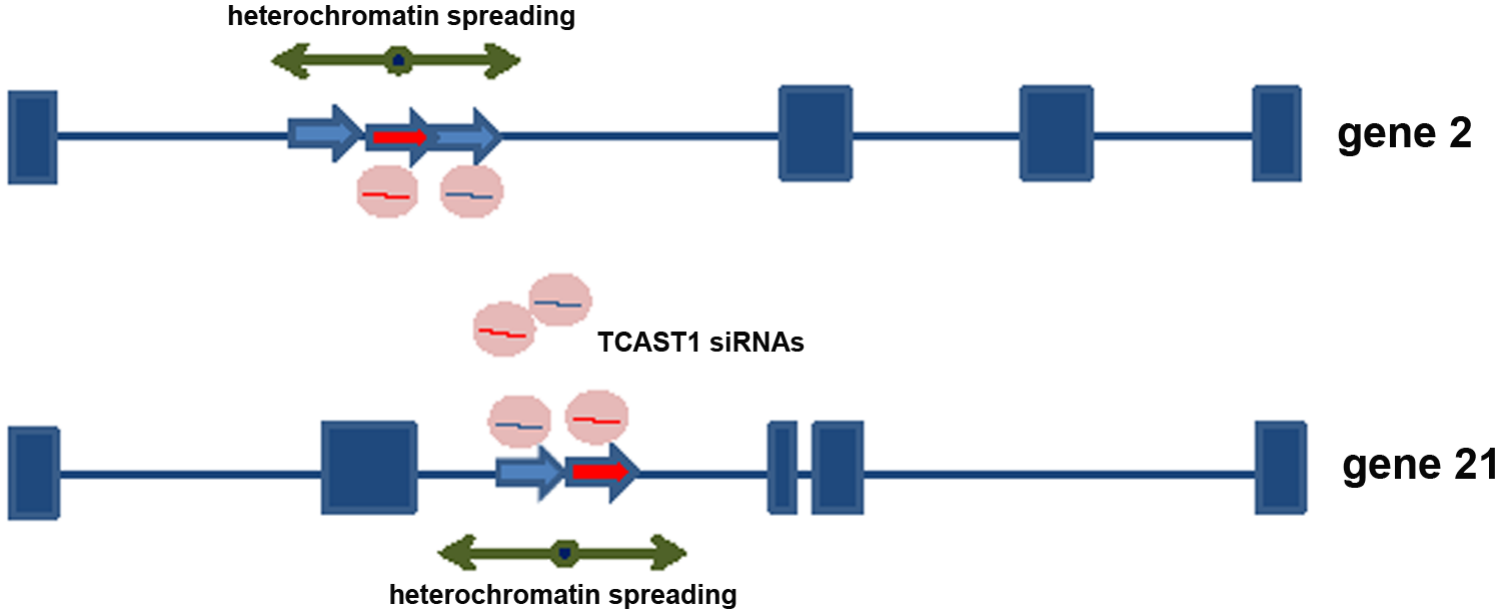
Model for satellite DNA- mediated suppression of genes. Transcripts of TCAST1 satellite DNA, specifically induced after heat stress, are processed into siRNA which target via sequence identity dispersed TCAST1 elements associated with genes. By guiding chromatin modifiers TCAST1 siRNAs increase the level of silent heterochromatin marks at dispersed TCAST1 elements and proximal regions. Temporary assembly and spreading of heterochromatin suppresses activity of nearby genes. Genes associated with elements 2 and 21 are schematically shown: exons are represented by rectangles, elements TCAST1 by arrows and complex containing TCAST1 siRNAs by a circle.

Previous investigation revealed a transient increase of TCAST1 satellite DNA transcription after heat stress which correlated with increased di-and tri- methylation of H3K9 at TCAST1 satellite DNA regions [22]. Now we propose that TCAST1-derived siRNAs target, via sequence identity, not only tandemly repeated TCAST1 satellite DNA within heterochromatin but also dispersed TCAST1 elements within euchromatin. Therefore, an increased level of TCAST1 siRNAs might possibly affect H3K9 methylation not only at tandemly repeated TCAST1 arrays within heterochromatin but also at dispersed TCAST1 elements and their flanking regions. Such “heterochromatinization” at dispersed elements and the spreading of heterochromatin marks to the neighbouring regions might consequently influence expression of nearby genes (Fig 5). In order to test this model we performed analysis of H3K9me2 and H3K9me3 levels within regions flanking dispersed polymorphic TCAST elements 2, 21 and 46 in different *T*. *castaneum* strains subjected to heat stress, using a chromatin immunoprecipitation (ChIP) assay. The H3K9me2/3 was analysed within regions 100–150 bp long, positioned within a few kb either 5’ or 3’ from the insertion site of TCAST1 elements. The position of primers used in ChIP experiments is indicated on S1 Fig. ChIP assay was performed on chromatin isolated from adult beetles subjected to long-term heat shock of 24 hr at 40°C, and the level of H3K9me3 and H3K9me2 was measured after 45 min and 1.5 hrs of recovery at 25°C and compared to the level of control (no HS) using the unpaired t-test. The level of H3K9me2/3 was also examined at TCAST1 repeats after long-term heat stress revealing a 100% and 80% increase of H3K9me3 after 45 min and 1.5 hrs of recovery, respectively, while in the same recovery periods the increase of H3K9me2 was lower, 30% and 50% respectively (S4a Fig).

**Fig 6:**
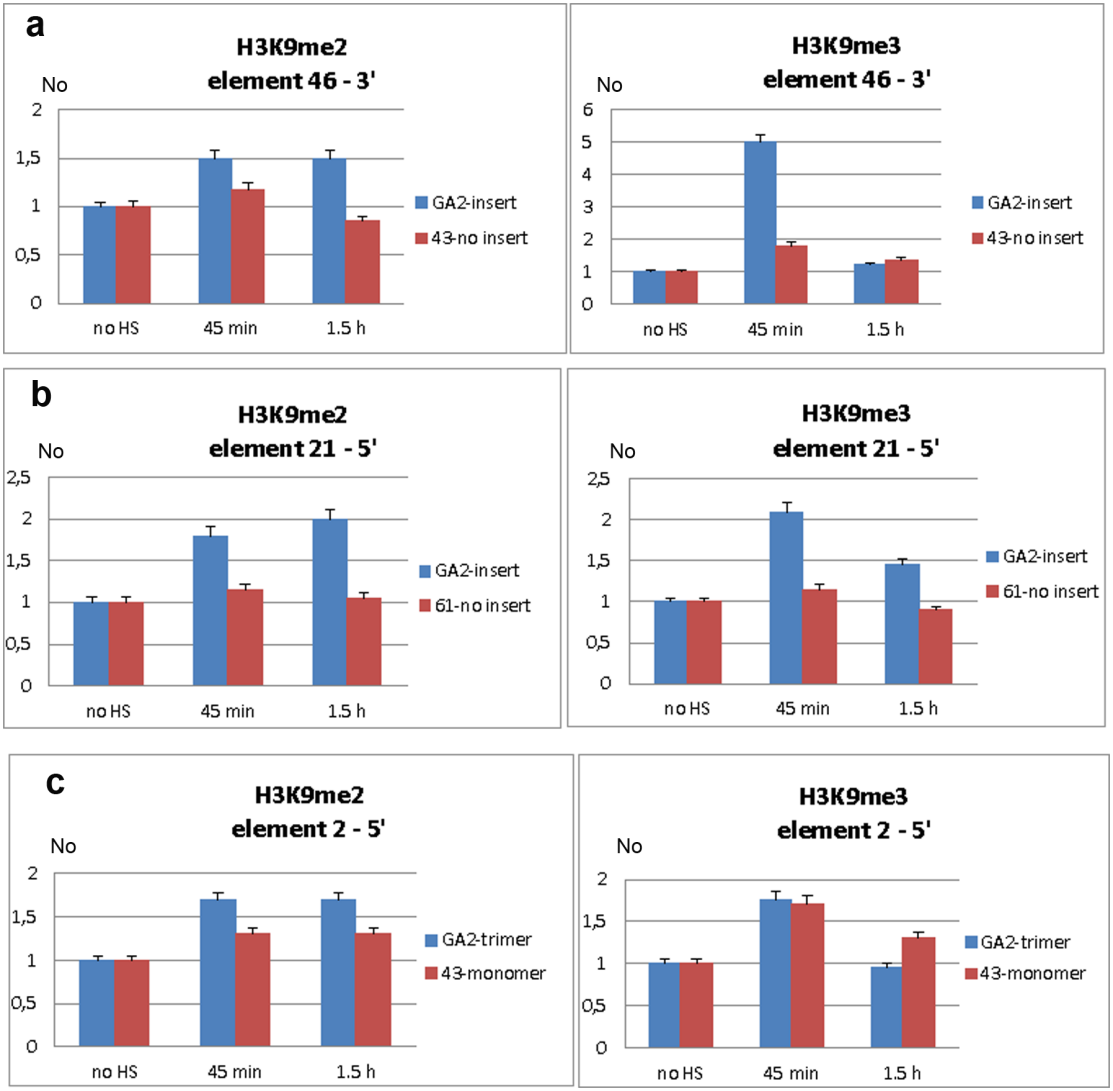
Analysis of H3K9me2 and H3K9me3 levels within regions flanking dispersed polymorphic TCAST1 elements. Level of H3K9me2 and H3K9me3 within region proximal to: **a)** element 46 in strain GA2 (blue) and within the same region in strain 43 (red) without element 46, **b**) element 21 in strain GA2 (blue) and within the same region in strain 61 (red) without element 21, **c)** element 2 in strain GA2 (blue) with element 2 in the form of trimer and within the same region in strain 43 (red) with element 2 as a monomer. H3K9me2/3 levels were measured by ChIP at standard conditions (no HS) and at 45 min and 1.5 hours of recovery period after long-term heat stress. Data show average of three independent replicate experiments and error bars indicate the standard error.

At a region 6.5 kb distant from the 3’ end of TCAST1 element 46 in strain GA2 the level of H3K9me2 was increased by approximately 50% at 45 min after heat stress and remained constant during the recovery period of up to 1.5 hrs (Fig 6a; P = 0.0260). The level of H3K9me3 was almost 5 times higher relative to the control (no HS) after 45 min of recovery (P<0.001) and dropped to the level of control after 1.5 hrs (Fig 6a). The corresponding region in strain 43 which does not have an inserted TCAST element 46, was characterized with no significant change in the level of H3K9me2 (P = 0.4374) after HS and with only slight increase in H3K9me3 (P = 0.0709) after 45 min of recovery (Fig 6a). In strain GA2, within a region at the 5’ end of TCAST1 element 46, a pattern of change of H3K9me2 and H3K9me3 after heat stress was very similar to the pattern observed at the 3’ site, while the same region in population 43 revealed no considerable change (S4b Fig).

At a region located 400 bp from the 5’ end of TCAST1 element 21 in strain GA2 the level of H3K9me2 was increased 45 min after HS, reaching a maximal two-fold increase relative to the control (no HS) after 1.5 h of recovery (Fig 6b; P = 0.0053). The level of H3K9me3 was also increased but with different dynamics reaching a maximal two-fold increase after 45 min of recovery and 50% increase after 1.5 hrs (Fig 6b; P = 0.0018). The corresponding region in strain 61 which does not contain a TCAST1 element, revealed no significant increase in H3K9me2 (P = 0.1474) as well as in H3K9me3 (P = 0.3844) relative to the control (Fig 6b). A region 800 bp distant from the 5’ end of TCAST1 element 2 in strain GA2 revealed an increase of 70% in the level of H3K9me2 at 45 min of recovery which remained constant up to 1.5 hrs of recovery (Fig 6c; P = 0.009). The same region in strain 43 where element 2 is in the form of a monomer showed a lower increase of 40% which was also constant during the recovery period (Fig 6c; P = 0.9359). The level of H3K9me3 at a region proximal to element 2 in both strains was similar and reached a maximal increase of 80% relative to the control after 45 min of recovery (P = 0.009 for trimer, P = 0.0144 for monomer) and after 1.5 hrs dropped to the level close to the control (Fig 6c).

The results reveal that exposure of adult beetles to heat shock is characterized by the enrichment of H3K9me2 and H3K9me3 at regions flanking both 5’ and 3’ to dispersed TCAST elements. The H3K9me2/3 increase was detected at regions within 1 kb distance from dispersed TCAST1 elements as well as at a region distant 6.5 kb. However, the same regions in strains which do not have TCAST1 elements inserted in the vicinity, do not exhibit a significant change in the two histone marks after long-term heat stress treatment. This reveals an essential role of dispersed TCAST1 elements in the accumulation of H3K9me2/3 marks and their spreading to proximal regions after heat stress. The peak of H3K9me3 was observed after approximately 45 min of recovery period and dropped down after 1.5 hrs of recovery to a level similar to that of the control, while the level of H3K9me2 remained increased after 1.5 hr of recovery. The dynamics of change of the specific heterochromatin marks H3K9me2 and H3K9me3 within regions proximal to dispersed TCAST elements is consistent with the observed change in the expression of a neighbouring gene: maximal increase of both histone marks is detected within the first hour after heat stress which coincides with the maximal repression of a neighbouring gene after 1 hr of recovery. After 1.5 hrs of recovery H3K9me3 is restored close to the control level, while H3K9me2 still remains increased resulting in approximately 80% of recovery of gene expression. The results indicate that satellite DNA-associated siRNAs, transiently activated after heat shock, affect the epigenetic state of dispersed TCAST1 satellite elements inducing their „heterochromatinization“. In addition, heterochromatin marks are spread to the regions flanking dispersed satellite DNA elements. The transient increase in H3K9me3/2 levels induces partial repression of genes located in the vicinity and confirms the proposed model of gene regulation mediated by satellite DNA (Fig 5).

## Discussion

Our present study shows that increased transcription of abundant TCAST1 satellite DNA in the beetle *T*. *castaneum*, which occurs after long-term heat stress, correlates with an increased level of repressive heterochromatin marks H3K9me2 and H3K9me3 not only on satellite repeats in constitutive heterochromatin but also on dispersed TCAST1 elements located in euchromatin. A temporary increase of heterochromatic marks on dispersed TCAST1 elements and their flanking regions correlates with the suppression of activity of nearby genes. This is the first report on satellite DNA elements influencing expression of nearby genes and confirms our previous hypothesis regarding the gene-regulatory role of satellite DNAs [4]. Aside from satellite DNAs, there are many studies on transposons which affect expression of neighbouring genes by spreading heterochromatin marks, as reported for diverse plants [25–27] and mammals [28]. In *Drosophila*, transposable elements dispersed within euchromatin were shown to trigger formation of H3K9me3 islands which are established through the action of the piRNA pathway, and influence expression of neighbouring genes [29]. In *T*. *castaneum*, the effect of dispersed TCAST1 satellite elements on nearby genes was not detected under standard physiological conditions as well as after short-term heat stress, although the short-term heat stress induces increase of TCAST1 siRNA levels [22]. However, restoration of the constitutive level of TCAST1 transcripts occurs at 30 min after short-term heat stress while after long-term heat stress the restoration takes approximately 3 hours. Long-term heat stress also results in complete cytosine demethylation of TCAST1 satellite DNA, while demethylation after short-term heat shock is only partial [30]. It could be proposed that increased TCAST1 satellite DNA expression and accumulation of TCAST1 siRNAs which occurs during a few hours after long-term heat stress is a prerequisite for the establishment of epigenetic changes on dispersed TCAST1 elements and their flanking regions in the form of H3K9me2/3 enrichment. This results in the repression of a proximal gene which occurs regardless of TCAST1 insertion upstream, downstream or within the intron and the level of repression seems to be constant up to a distance of 40 kb from the transcription start site (TSS). However, it cannot be excluded that in addition to H3K9me2/3 enrichment some other epigenetic changes occur within the same chromatin regions after long-term heat stress and also affect activity of nearby genes.

TCAST1 satellite DNA repeats dispersed within euchromatin are distributed equally on all chromosomes [8]. Analysis of distances of TCAST1 elements from TSS of associated genes based on published data [8], shows that 70% of them are within range of 40 kb, while only 10% are above 100 kb (S5 Fig). This indicates that most of these TCAST1 elements are located within distances at which their effect on the expression of neighbouring genes is maximal. Such a distribution of elements with the majority of them influencing genes may also indicate that the effect of TCAST1 on genes is probably not deleterious, but may be either neutral or beneficial. Polymorphism analysis of 32 TCAST1 elements, 19 transposon-like and 13 satellite-like among ten populations revealed insertion polymorphism in four satellite—like elements while transposon-like elements were fixed at 100%. Differences in the pattern of distribution of TCAST1 elements contribute to gene expression diversity among *T*. *castaneum* populations and might have some impact on adaptation of populations to different environmental conditions. Geographical populations of *D*. *melanogaster* exhibit variation in thermal sensitivity which is partially due to regulatory variation exerted by repetitive elements abundant in the Y chromosome [31].

What might be the physiological consequence of the temporary change in activity of TCAST1-associated genes after long-term heat stress? In order to answer this question, it would be necessary to understand the function of TCAST1-associated genes and the biological processes in which they are involved. Although many genes in *T*. *castaneum* are not well annotated, previous analysis of TCAST1-associated genes showed a statistically significant overrepresentation of immunoglobulin-like genes within this gene set [8]. The molecular function of most of these immunoglobulin-like genes is unknown and they might be involved in different biological processes such as cell adhesion, protein phosphorylation and axon guidance. Based on iBeetle-Base (http://ibeetle-base.uni-goettingen.de/) [32] which contains RNAi phenotypes in *T*. *castaneum*, knock down of almost 45% of TCAST1-associated genes causes severe developmental defects either in embryogenesis or metamorphosis and affects survival. It is therefore very probable that the modulation of activity of TCAST1-associated genes induced by environmental factors such as long-term heat stress might influence development of *T*. *castaneum*. It is also important to mention that since different TCAST1- associated genes are modulated in parallel, their effect on biological processes could be cumulative.

It can be also hypothesized that the temporary suppression of TCAST1-associated genes after long-term heat shock might influence stress recovery process. Heat shock is a substantial stress to cells which immediately respond by activation and expression of heat shock proteins (HSPs) that act as molecular chaperons involved in restoration and maintenance of proteins [33]. Another possible response to heat stress might be downregulation of expression of most of genes except *HSPs* which is observed after heat stress, presumably leading to the reduction of damaged proteins in heat stressed cells [34]. Since heat stress provokes a number of remarkable changes inside the cell including massive destruction and reorganization of cellular components [35], the process of cell recovery after heat stress is complex and includes sequential restoration of gene expression. We propose that dispersed TCAST1 satellite DNA elements, by modulating the activity of the neighbouring protein-coding genes within a short time frame after long-term heat shock, might contribute to the recovery of cells and organism from harmful heat-stress conditions.

Further studies are necessary to see if transient suppression of genes by dispersed satellite DNA elements is a general phenomenon or it is only limited to TCAST1 elements which are very abundant within pericentromeric heterochromatin and whose expression within heterochromatin is significantly affected by heat stress. To answer this question other minor satellites in *T*. *castaneum* will be examined and their effect on gene expression checked as well as satellite DNAs in other model organisms.

## Materials and Methods

### Beetle strains

The following strains of *T*. *castaneum* were used: **GA2** strain, originally used in the genome sequencing project and deriving from North American wild-type strain collected in Georgia in 1982, obtained from Dr. Dick Beeman (Manhattan, KS, USA) and wild-type strains: **GA1**- collected in Georgia, USA in 1980; **43**—collected at Kyushu Island, Japan in 1988; **50**—collected in Schegel Farm, Indiana, USA in 2005; **51**—collected in Adrian, Missouri, USA in 2006; **52**—collected in Bloomington, Indiana, USA in 2006; **55**—collected in Jerez, Spain in 1991; **57**—collected in Perù, in 2002; **61**—collected in Banos, Ecuador in 1996; **Zg** collected near Zagreb (Božjakovina), Croatia in 2010 and **VT** collected at Veliko Trgovišće, Croatia in 2010. Each laboratory stock was established and maintained as a separate culture at a population size of >200 individuals on standard medium (20: 1, flour: brewer’s yeast, by weight) in a dark incubator at 24°C and approximately 70% relative humidity. Heat shock treatment of adult insects was done at 40°C for 24 hrs under 70% relative humidity.

### DNA extraction and PCR analysis

DNA was extracted from 50 to 100 mg of adult insects, which corresponds to 10–20 individuals, using the DNeasy Blood & Tissue Kit (Qiagen). DNA concentration was measured using the Quant-iT dsDNA assay kit and the Qubit fluorometer (Invitrogen) and the average amount of DNA isolated using the DNeasy kit ranged from 30 ng/μl to 60 ng/μl. Primers used in the analysis of polymorphism of 32 TCAST1 elements where designed in a unique sequence around the insert (upstream and downstream from insert) using Primer3Plus software and are listed in S1 Table. It was not possible to design primers for 36 TCAST1 inserts due to poor annotation in GenBank (unique sequence necessary for designing primers was missing) or enrichment of neighbouring sequence with repetitive elements other than TCAST1 elements. PCR reactions were performed using premade 2 x DreamTaq Green PCR Master Mix (Fermentas) in a final reaction volume of 20 μl containing 0.2 μM of each primer and 50 ng of genomic DNA. The following reaction conditions were used for the amplification: 94°C for 1 min, 10 cycles at 94°C for 30 s, 60°C for 30 (with annealing temperature declining in each cycle for 0.25°C) and 72°C for 1 min followed by 20 cycle at 94°C for 30 s, 55°C for 30 s and 72°C for 90 s. PCR amplification products were subjected to 1.2% (w/v) agarose gel electrophoresis and if PCR product size differed from the expected one (insert was absent or present in different copy number), a band was cut from the gel, DNA isolated using QIAquick gel extraction kit and sequenced using an ABI Prism 310 (Applied Biosystems).

### RNA isolation and reverse transcription

RNA used in the analysis of gene expression was isolated from *Tribolium* adult beetles (approximate weight of sample 20 mg) using the RNeasy Mini kit (Qiagen) according to the manufacturer’s instructions. In strain 57, due to individual polymorphism of TCAST1 element 2, RNA was isolated from single individuals. RNA was additionally digested with Turbo DNase (Ambion), quantified with the Quant-IT RNA assay kit using Qubit fluorometer (Invitrogen), quality-checked on gel, and PCR-checked for the presence of TCAST DNA. RNA (approximately 1 μg) was reverse transcribed using PrimeScript RT-PCR Kit (Takara) in 10 μl reaction using random primers. Negative controls without reverse transcriptase were used for all samples.

### Quantitative real-time PCR (qPCR) analysis

cDNA samples were amplified using Applied Biosystem ABI7300 Real-Time PCR System. The qPCR reactions were done in triplicate, in 50 μl reaction volume with 0.5 μM specific primers, 2X Power SYBR Green PCR Master mix (Applied Biosystems) and 30 ng of cDNA. Primers for expression analysis of TCAST1 associated genes are listed in S1 Table, while primers for expression analysis of TCAST1 satellite DNA are: F CCATAAGCGAGTTATAGAGTTGG and R CTTTAGTGACTTTTATGTCTTCTCC. To correct for the differences in sample composition and in the yield of the reverse transcription reaction, ribosomal protein S18 (RPS18) was used for normalization [36, 37]. The thermal cycling conditions were as follows: 50°C 2 min, 95°C 7 min, 95°C 15 s, 60°C 1 min for 40 cycles followed by dissociation stage: 95°C for 15 s, 60°C for 1 min, 95°C for 15 s and 60°C for 15 s. TaqMan probe for gene associated with element 21 as well as TaqMan Gene Expression Assay were obtained from Applied Biosystems. Amplification specificity was confirmed by dissociation curve analysis. Specificity of amplified product was additionally tested on agarose gel. Control without template (NTC) was included in each run. Post-run data were analysed using LinRegPCR software v.11.1. [38, 39]. The software enables calculation of the starting concentration of amplicon (“No value”) which is expressed in arbitrary fluorescence units and is calculated by taking into account PCR efficiency and baseline fluorescence. “No value” determined for each technical replicate was averaged. Averaged “No values” for gene of interest were divided by “No values” of endogenous control for normalization. Statistical analysis of qPCR data between populations was done using GraphPad v.6.01. Normalized No values were compared using unpaired t-test which compares the mean of two unmatched groups. Strain variations were analysed using one-way ANOVA test.

### Chromatin immunoprecipitation

Adult beetles were frozen and homogenized in Nuclear Isolation buffer (10 mM MOPS; 5 mM KCl; 10 mM EDTA; 0.6% Triton X-100) containing protease inhibitor cocktail Complete Mini (Roche). Un-homogenized tissue was removed by centrifugation at low rpm for 1 min at 4°C. Formaldehyde in a final concentration of 1% was added to supernatant, followed by incubation for 10 min at RT and addition of 0.136 M glycine. Nuclei were pelleted by centrifugation, resuspended in Nucler Lysis buffer (50 mM Tris-HCl pH 7.7; 1% SDS; 20 mM EDTA) and sonicated 8 times for 20 s on ice, centrifuged at max speed for 3 min at 4°C. 10% of chromatin was stored for input fraction. The remaining supernatant was incubated for 3 h at 4°C with pre-prepared solution of Protein A-coated paramagnetic beads (Dynabeads® Protein A, Invitrogen) and antibodies in ChIP dilution buffer (1% Triton X-100; 10 mM EDTA; 15 mM Tris-HCl pH 7.5; 300 mM NaCl). The immune complex was subsequently washed five times in ChIP dilution buffer, beads were captured by magnet and the immuno-complex was eluted by centrifugation for 10 min at 65°C in elution buffer (1% SDS; 10 mM NaHCO_3_). The reversal of cross link was performed by addition of 20 mM EDTA and 0.2 M NaCl and incubation overnight at 65°C. The material was digested with proteinase K (200 μg/ml) and DNA was extracted by phenol: chloroform: isoamyl alcohol (25:24:1). The following antibodies were used: H3K9me3 (Abcam, ab8898) and H3K9me2 (Abcam, ab1220). Binding of precipitated target was determined by qPCR using the SYBR Green PCR Master mix, primers listed in supplementary S1 Table and reaction conditions described above. The No value was normalized using No value of input fraction.

## Supporting Information

S1 Fig Schematic representation of genes associated with TCAST1 elements 2, 12, 21 and 46. Exons are represented by rectangles, TCAST1 elements by blue (Tcast1a) and red (Tcast2b) arrows. Yellow arrows indicate positions of primers used for gene expression analyses, while pink arrows show positions of primers used in ChIP experiments.
(TIF)

S2 Fig Expression of genes associated with TCAST1 elements 2 and 46 in GA2 strain at standard conditions (no HS) and after one hour of recovery following short-term heat stress for 2 hrs (2h HS) and for 8 hrs (8h HS) at 40°C. No significant change in the expression of both genes at standard and short-term heat stress conditions was detected (P>0.200).
(TIF)

S3 Fig Expression of TCAST1 satellite DNA in strains GA2 and GA1 under standard conditions (no HS) and after one hour of recovery following long-term heat stress at 40°C (HS 1h). The level of transcripts is increased 3x (P<0.001) in both strains after long-term heat stress.
(TIF)

S4 Fig Analysis of H3K9me2 and H3K9me3 levels within: a) pericentromeric and dispersed TCAST1 regions in strain GA2, b) a region at 5’ end of TCAST1 element 46 in strains with (GA2) and without (43) element 46. Levels of H3K9me2/3 were measured at standard conditions (no HS) and at 45 min and 1.5 hours of recovery period after long-term heat stress using ChIP. Significant increase of H3K9me2/3 is detected at TCAST1 region (P<0.01) as well as at 5’ end of TCAST1 region in GA2 strain (P<0.02), while no significant change is detected in strain 43 (P>0.1). Data show average of three independent replicate experiments and error bars indicate the standard error.
(TIF)

S5 Fig Distribution of distances of dispersed TCAST1 elements relative to the transcription start sites of the associated genes. Number of distances of dispersed TCAST1 elements within 100 kb from transcription start sites (TSS) are shown.
(TIF)

S1 Table List of primers used for the analysis of polymorphism of dispersed TCAST1 elements, for the expression analysis of TCAST1-associated genes and for the analysis of histone methylation within regions flanking polymorphic TCAST1 elements. (DOCX)
