# Correction: Satellite DNA Modulates Gene Expression in the Beetle *Tribolium castaneum* after Heat Stress

**DOI:** 10.1371/journal.pgen.1005547

**Published:** 2015-09-25

**Authors:** Isidoro Feliciello, Ivana Akrap, Đurđica Ugarković

In the legends for Fig 1 and S1 Fig, the name of the TCAST1 element subfamily Tcast2b is incorrect and should be replaced with Tcast1b. The correct legends are below.

**Fig 1:**
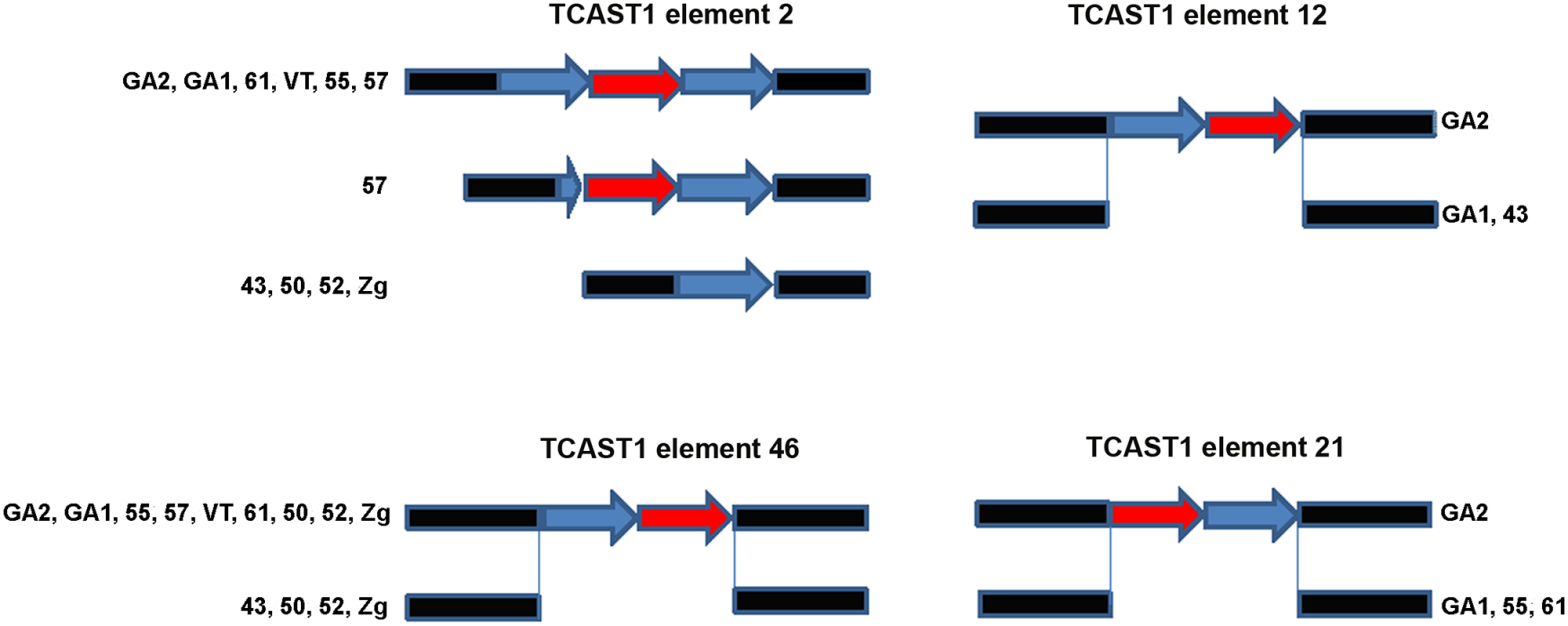
Schematic representation of dispersed TCAST1 elements 2, 12, 46 and 21 in *T*. *castaneum* strains. Blue and red arrows represent repeats belonging to subfamilies Tcast1a and Tcast1b, respectively, while flanking sequences are shown in black.

In the Author Contributions section, Isidoro Feliciello (IF) and Ivana Akrap (IA) should be listed in the section “Wrote the paper”. The correct contributions are: Conceived and designed the experiments: IF ĐU. Performed the experiments: IF IA. Analyzed the data: IF IA ĐU. Contributed reagents/materials/analysis tools: ĐU IF. Wrote the paper: IF IA ĐU.

## Supporting Information

S1 Fig Schematic representation of genes associated with TCAST1 elements 2, 12, 21 and 46. Exons are represented by rectangles, TCAST1 elements by blue (Tcast1a) and red (Tcast1b) arrows. Yellow arrows indicate positions of primers used for gene expression analyses, while pink arrows show positions of primers used in ChIP experiments.
(TIF)
