# Supplementary figures and images for: Satellite DNA Modulates Gene Expression in the Beetle Tribolium castaneum after Heat Stress
Source: PLoS Genet. 2015 Aug 14;11(8):e1005466. doi: 10.1371/journal.pgen.1005466 (PMC4537270; doi:10.1371/journal.pgen.1005466)

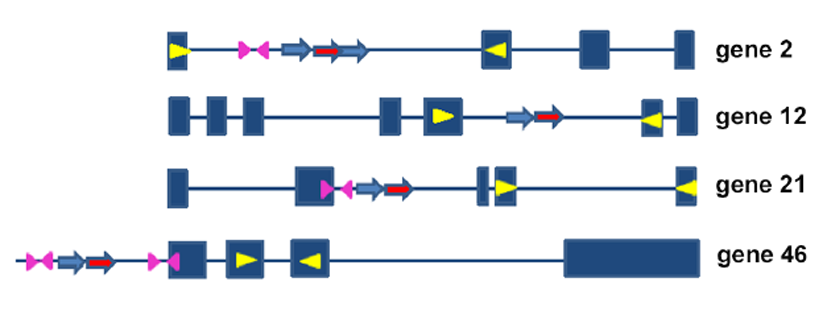

Supplement: S1 Fig — Exons are represented by rectangles, TCAST1 elements by blue (Tcast1a) and red (Tcast2b) arrows. Yellow arrows indicate positions of primers used for gene expression analyses, while pink arrows show positions of primers used in ChIP experiments. (TIF) [file pgen.1005466.s001.tif]

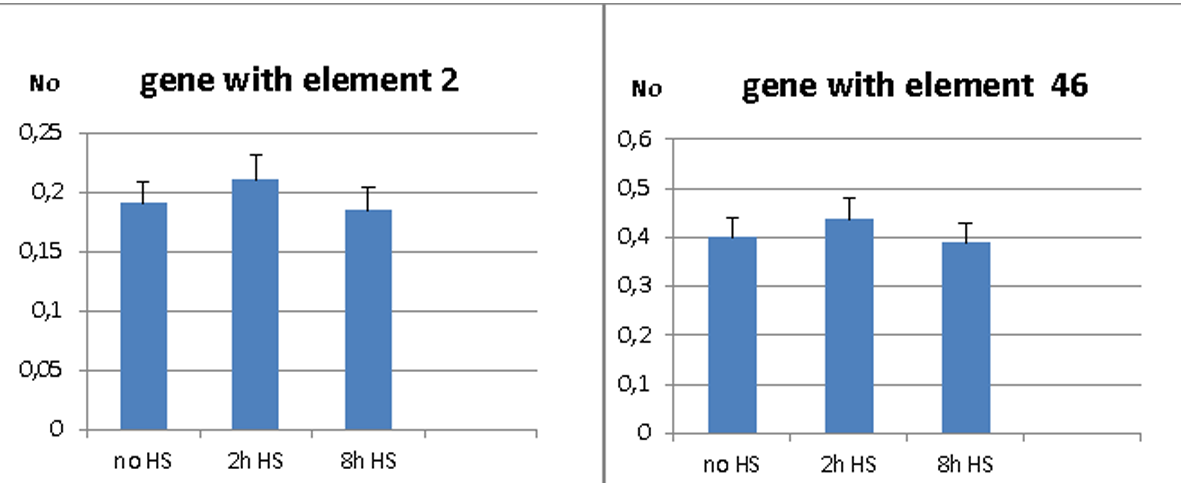

Supplement: S2 Fig — No significant change in the expression of both genes at standard and short-term heat stress conditions was detected (P>0.200). (TIF) [file pgen.1005466.s002.tif]

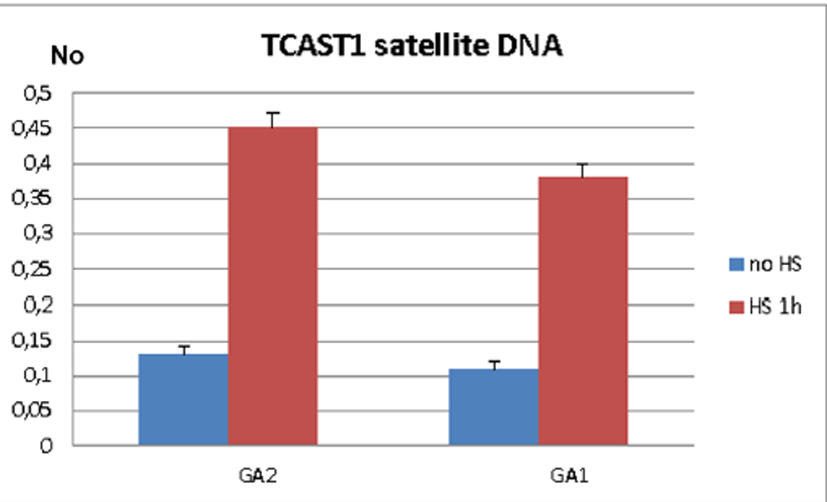

Supplement: S3 Fig — The level of transcripts is increased 3x (P<0.001) in both strains after long-term heat stress. (TIF) [file pgen.1005466.s003.tif]

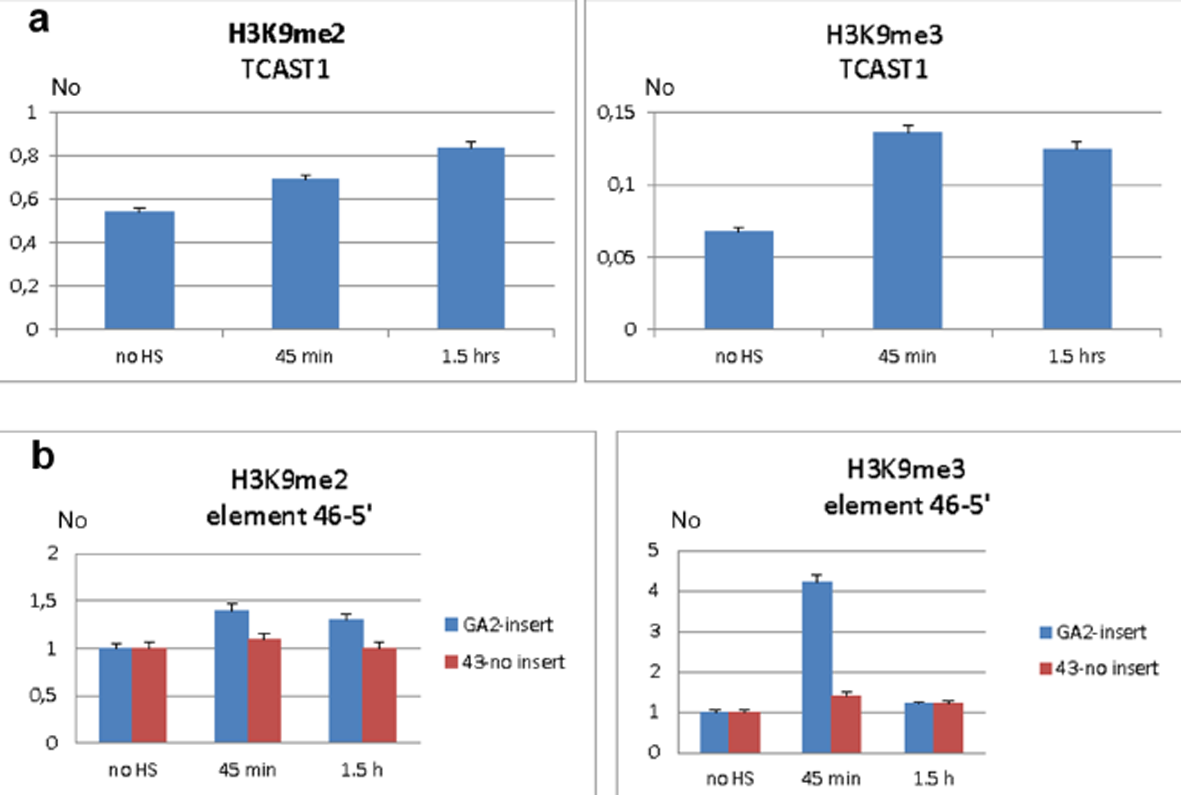

Supplement: S4 Fig — Levels of H3K9me2/3 were measured at standard conditions (no HS) and at 45 min and 1.5 hours of recovery period after long-term heat stress using ChIP. Significant increase of H3K9me2/3 is detected at TCAST1 region (P<0.01) as well as at 5’ end of TCAST1 region in GA2 strain (P<0.02), while no significant change is detected in strain 43 (P>0.1). Data show average of three independent replicate experiments and error bars indicate the standard error. (TIF) [file pgen.1005466.s004.tif]

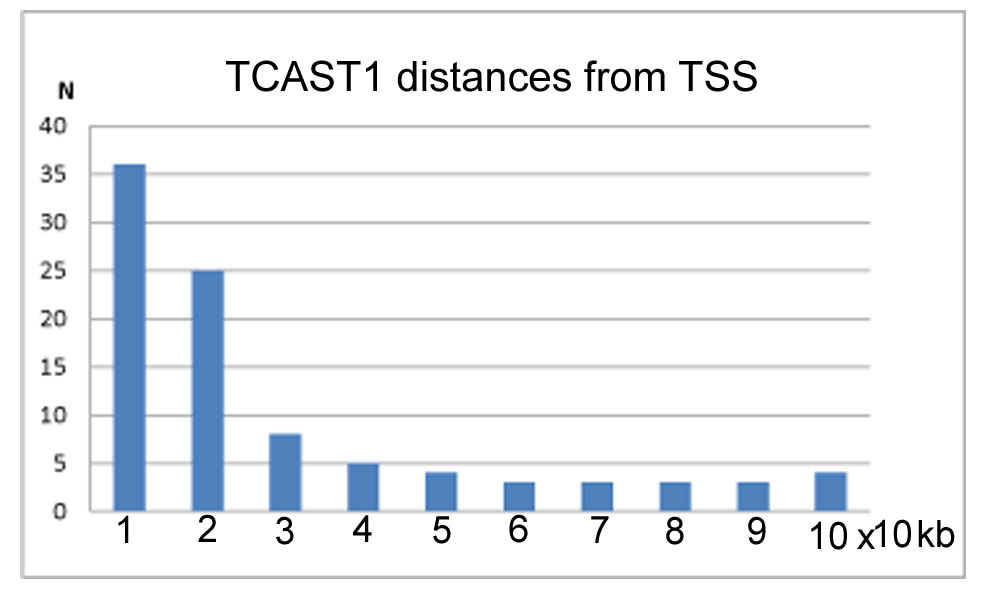

Supplement: S5 Fig — Number of distances of dispersed TCAST1 elements within 100 kb from transcription start sites (TSS) are shown. (TIF) [file pgen.1005466.s005.tif]
