# Supplementary material for: Satellite DNA Modulates Gene Expression in the Beetle Tribolium castaneum after Heat Stress
Source: PLoS Genet. 2015 Aug 14;11(8):e1005466. doi: 10.1371/journal.pgen.1005466 (PMC4537270; doi:10.1371/journal.pgen.1005466)
Supplement: S1 Table — (DOCX) [file pgen.1005466.s006.docx]

**S1 Table:**

List of primers used for the analysis of polymorphism of dispersed TCAST1 elements, for the analysis of expression of TCAST1-associated genes and for the analysis of histone methylation within regions flanking polymorphic TCAST1 elements.

| TCAST1 element | Primers for polymorphism analysis | | Primers for expression of TCAST1 associated genes | | Primers for ChIP |
| --- | --- | --- | --- | --- | --- |
| 2 | F | GATGCACCTTGTTCGCACCTTTG | F | CCAGACCATTTCGAGGATGT | Up: CACGTCGTAGACAAGGCATTAG |
|  | R | AGGTCATGGCTTCCTAGCACCG | R | TTTATCGACGGAACGGACTC | Up: AGAATCGCTCGTCTTTGTGTG |
| 4 | F | GAGAGCTTCTGTTTGGCATTAG | F | *Pseudogene |  |
|  | R | CCTGAAATACCGACACGAAAG | R | / |  |
| 9 | F | AAACACGTGCTAAAAGGGCTGAC | F | TGGTGGTCAGTGTCAAGGATCT |  |
|  | R | CAAACGCTCGTTCAAATGCTTGC | R | CAGGAGGGAGTGGTTGTCTTC |  |
| 10 | F | CATCCCAAACAGGCAAGATAA | F | TTCCTAGTTTATGAGGGCTTGATG |  |
|  | R | TGGAGATCATGCGTTTACATTC | R | TCCGAATTGCCATAGGATTGTC |  |
|  |  |  | F | ATGACAAAAGGGCGTGCTAC |  |
|  |  |  | R | GGCTGCATTCTCTCTGAACAT |  |
| 11 | F | GCTTTAACGTGCTTTAGGACAA | F | GTACCATATCCAGCAAGAAGTGAAC |  |
|  | R | GCTCGAAATGAAACAGGAATAG | R | ATGTCCTTCTTCGTCCAACAGA |  |
| 12 | F | CAGATTTCATGGAACTCATGGGC | F | GGCTGCTTCGAGGATATTAAAG |  |
|  | R | CCGGAAGAATTCAGAATACAGAAAGC | R | CAAAGCCATACATTTTCCTTCA |  |
| 13 | F | TGACCATTGACTATTGACTACGGA | F | CAGCGAGCATATTAAACCTGTG |  |
|  | R | CTGCCTGTATTACACAATTTCAACC | R | AATGGGAACGAACGATATAAGC |  |
| 14 | F | GACTACCGAGAATATCTGTTCACAG | F | AGACATTCTTGCACACCCGA |  |
|  | R | GAGGATCGATTACGGCCGTAT | R | AACTCCATACCCACCCTTGAC |  |
| 15 | F | ATGCAAAGAGTACATGCTGTGA | F | CTGTGCCATAATCCACCACTT |  |
|  | R | CGACCATCTAACACGACAAGAA | R | GACGGAACATTCGACCATATCT |  |
| 17 | F | CGTCCGATTTACACTCAAACTCAC | F | GCCTGGTTATTACTGGAGGTTCT |  |
|  | R | ATTAAACCGATTGAGAGAGGTTGGT | R | GGTTGAGGGTAACACGGATTT |  |
| 21 | F | GCTCGTCACTCCAATTAAGGTACA | F | CAGTTATGGTGCGAGTGTGTTT | Up: TTCTCTGTACCCTTCGAGCAA |
|  | R | CTGGTCAAAAGGCCGAATATAA | R | GGAAAAGAACGTCATCCGAGTA | Up: ATTGGAGTGACGAGCATGTG |
| 23 | F | CTTTGGATGGGTGTGGTAATCT | F | ACTGACCCGAATACAGCTTTTCT |  |
|  | R | AAACGTGCAGATACTGTGGTTG | R | GCACCTCGTTTTAGCAGTTCTC |  |
| 25 | F | ATTAGTGGTGATCGCACACG | F | ACCTGTGGCTTTCAGTTTAGTTG |  |
|  | R | GCTAGATGGGAAACGTAAGAC | R | CACCTCCACTACAAACCTTGAAC |  |
| 26 | F | AGGCTTCGAGTTTCTCCTTGT | F | TTGAAGCTGGAGGATATGGAAAC |  |
|  | R | AAGTGTCGATTTGTCCCAAGAC | R | CTAAGCCGTTGATTAGGGTTGAG |  |
| 27 | F | GAACGAATCCTCACAAGGCTAC | F | GCAAGGTAGACTGATTTTGAGAGA |  |
|  | R | CGCAAGTGTTTTGAAGACCAG | R | CCGATTGTGTAAGATTCCATGA |  |
| 28 | F | GTGTTCCTTTCGTGTCACACC | F | *Pseudogene |  |
|  | R | GCAATAATTGTGCCGAATGATACG | R |  |  |
| 30 | F | GTCCAAGATTAACCACTTGATACC | F | TAGTGCAACTGAGGTGGCTT |  |
|  | R | ATTGCATGTCGAAGCTGTTG | R | AAGCGCAAGTGGAGGTATTTC |  |
| 32 | F | CCGTCGCGTAATGGCTGCGA | F | TGCGCTGGTTATACTCGTCTT |  |
|  | R | GCACACCCTTTGAGGTTCTGCCA | R | CCAGTTTCAAGTCGGAGATATTG |  |
| 33 | F | TGCAGATAGGAGGTGTTCTCAA | F | TGAAGAACTGAACGGCGATATT |  |
|  | R | GCCAAAGCCTATCCAACCTTAT | R | CCGGATTGATTGATTTGTTGAG |  |
| 34 | F | AGCCTAATTCGCAAGAACAGAC | F | GCTCATGGGAGTTTTTCAATGT |  |
|  | R | TCAGTCAGGTCAAGATCAGGTTT | R | GTATGAAGACGACAAATGCGAG |  |
| 38 | F | / | F | *Pseudogene |  |
|  | R | / | R |  |  |
| 39 | F | AATCCAAACGTCCATGCGTGTAT | F | GACAATATCACACGTCCCATTCA |  |
|  | R | CAAATAAGGCATTGTAATGGCGG | R | ATTCTAGCCACGCAACGTATTT |  |
| 40 | F | TCACCTGAGGACGACCACTTT | F | GGGCGAGTATGTTTGAATGTG |  |
|  | R | GGAAGATTTGGAGAGTGATACCGAT | R | TAGACGTGAACGGGATTAGTCA |  |
| 41 | F | CTCGCGGCTGTTAAGTGGC | F | CTCAACTGCCGAATCAGAAACT |  |
|  | R | CCGCGAAGAAATGCATGCGCT | R | TATGTGGATCTGCCCACTGTTA |  |
| 42 | F | GCTCGGCTATCCTTCTAGTTTG | F | GTACGCGAATGTCAACAGAAAG |  |
|  | R | AAATGGTAGCAGCGTTTCAACT | R | GAGCATTCCGCATCAAAGTC |  |
| 44 | F | AAGTGCTCGCAGTACCAAAAGT | F | AAGTGCTCGCAGTACCAAAAGT |  |
|  | R | CAGAAGCTGTAATTCCCCTAGAAA | R | TGATGTGATGGAAGGACACAAT |  |
| 46 | F | ACTCCCTGAGAAAAGTTGGAC | F | GAGTTTGTCGATCCTGTCTCAAT | Up:TCTCACCCCTTCGACTACACTT |
|  | R | ACCAAAGACCAGTGATTCTATCG | R | GCAATCCGAGCTGCAAGAATA | Up: GACACCCCTGTCCAACTTTTCT |
|  |  |  | F |  | Down: TAAACTGGGTAGCGCCCTCTT |
|  |  |  | R |  | Down:CTGAGTGTTTTCCCGCTCAAATA |
| 48 | F | ACTTCCTATGTTAGCCCAGTCAA | F | GTACGATGGGACGGCTACAG |  |
|  | R | CTCATCCTCCCACTTCTTCTGT | R | TCCTTGGACAAACCGACATAA |  |
| 52 | F | GCAGGAAGTCCCACAAAACTAC | F | GAAGCACTGTAAATGTCGATGTG |  |
|  | R | ACTAACCACCACGCATTCTTTC | R | AGTGGCTGCTGTTGCTTACA |  |
| 57 | F | CCGATTTTCCGTATCGTTTTAC | F | TTCCGCTCAGGACGAACTATAA |  |
|  | R | ATCCAGACGAAGATTATGCCAG | R | CAGACGAAGATTATGCCAGAGC |  |
| 58 | F | AATTCGGTTGAGGATGACAGTT | F | ATACAGTTTTGAGCACGAATGC |  |
|  | R | AGCAGCATGAATGTAGCATGAA | R | AGGCCAGTTTGGATTATTACCAC |  |
| 59 | F | GTGAGTAAGTGTGGCGTAATGTTT | F | TTTTCCCGAGCAGTTACAAGAC |  |
|  | R | CAACATTCCAGGTTTCTTTCAC | R | GATTCGGCACAGTTAAAACCAT |  |
| 67 | F | TTTTGTCCGCAGGTGTACTATC | F | ATAGTTCTAGCGACGGGATTGA |  |
|  | R | AGCTCGAAGAGGGGGAATAG | R | CCACTTCTTGCACCGACTTTAT |  |
