# Supplementary figures and images for: Correction: Satellite DNA Modulates Gene Expression in the Beetle Tribolium castaneum after Heat Stress
Source: PLoS Genet. 2015 Sep 25;11(9):e1005547. doi: 10.1371/journal.pgen.1005547 (PMC4583542; doi:10.1371/journal.pgen.1005547)

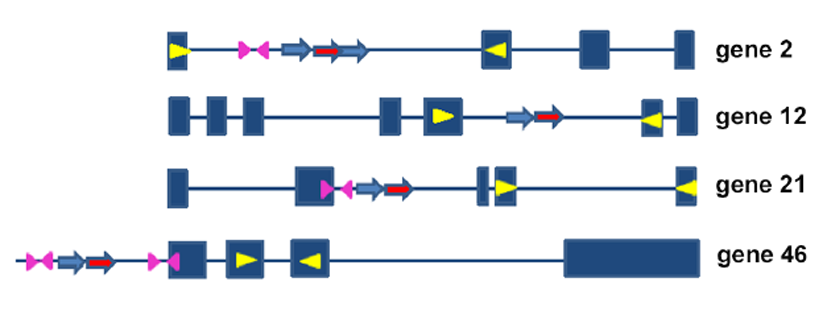

Supplement: S1 Fig — Exons are represented by rectangles, TCAST1 elements by blue (Tcast1a) and red (Tcast1b) arrows. Yellow arrows indicate positions of primers used for gene expression analyses, while pink arrows show positions of primers used in ChIP experiments. (TIF) [file pgen.1005547.s001.tif]
